# Polymorphism of Genes and Implantation Failure

**Published:** 2013

**Authors:** Majid Mojarrad, Mohammad Hassanzadeh-Nazarabadi, Niaiesh Tafazoli

**Affiliations:** 1 *Department Medical Genetics, School of Medicine, Mashhad University of Medical Sciences, Mashhad, Iran.*; 2 *Medical Genetics Research Center, School of Medicine, Mashhad University of Medical Sciences, Mashhad, Iran.*

**Keywords:** Gene, polymorphism, implantation failure

## Abstract

Implantation failure is the most frequent cause of pregnancy loss in couples who try to conceive, either in a natural way or using assisted reproductive techniques (ART). Identify the precise mechanisms of implantation failure can lead to identify couples at risk and also providing appropriate therapeutic options to affected couples. Despite the high prevalence of this disorder, a few causing factors are demonstrated so far. Recent studies indicate that genetic factors play an important role in the occurrence of recurrent implantation failure. Although some of these factors, such as numerical chromosomal aneuploidy are known to be causative factors, there are some other factors that solely increase susceptibility to this event. In the present review we try to list the genetic polymorphisms that are known as susceptibility factors in implantation failure.

Pregnancy loss can be caused by several factors which are involved in fundamental events during human reproduction. Anatomical, immunological, hormonal and infectious factors along with known genetic factors are involved in 50% of such cases. Our previous findings have shown that 9.8% of pregnancy loss suffering couples have chromosomal balanced rearrangement (1). Furthermore single gene disorders seem to be important factor in pregnancy loss, as our previous results show that there is a correlation between consanguineous marriage and the occurrence of idiopathic spontaneous fetal loss (2).

Since about 80% of pregnancies are lost during the first trimester, it has been postulated that the major cause of failed pregnancy is an error of embryo implantation (3).

Genetic factors that lead to implantation failure have overlap with those involved in recurrent spontaneous abortion and infertility (4-6).

Implantation failure is the most frequent cause of lack of pregnancy after in vitro fertilization (IVF) and embryo transfer, as implantation failure takes place in approximately 40% of IVF experiments (3). Successful implantation requires trophoblastic growth, invasion into the endometrium and stimulation of vascularization to provide its own blood supply (4-6).

There are increasing numbers of evidence indicating that genetic factors regulating invasion and angiogenesis processes are critical in embryo implantation. Genetic defect and even genetic polymorphisms of genes involved in these processes can lead, or at least increase susceptibility to implantation failure (4-6).

In the present review we will attempt to provide a list of research studies performed in this area and genetic factors which are involved in implantation failure.

**P53 tumor suppressor gene**

P53 is mostly known as a “genome guardian” who has a pivotal role in genome integrity maintenance and tumor prevention. P53 becomes activated by sense of wide variety of stress signals and initiate a transcriptional program leading to apoptosis, cell cycle arrest or senescence. P53 itself is under negative control of some genes such as Mdm2. By binding to the p53 protein, Mdm2 leads to p53 polyubiquitination and sending it to the proteosome (4-6).

Phylogenic analysis of P53 revealed that P53 is an evolutionary conserved gene and P53-like transcriptional factors exist in short - lived invertebrates that do not exhibit adult tumors. These findings suggest that P53 may have another initial role in these creatures.

Several studies performed in recent decade indicate that p53 has a critical role in maternal reproduction (7, 8). While p53 ^-/- ^male mice show normal reproduction rate, p53 ^-/- ^female mice show reduced pregnancy ability and litter size when mated with p53 ^-/-^_, _ p53 ^-/+ ^and p53 ^+/+^ male mice and worst pregnancy rate and litter size in mating with p53 ^-/-^ male mice (9). It has been suggested that P53 activates embryo implantation into the uterus as a stress signal and induces expression of several genes needed for initiation and establishment of embryo implantation.

According to the high penetrance of p53 mutations in tumor formation it was assumed that single nucleotide polymorphisms of p53 lead to infertility of clinically normal women. To date several functional SNPs have been identified in both p53 and its negative regulator, Mdm 2, which can alter the expression or function levels of p53 (10).

P53 codon 72 single nucleotide polymorph-ism is one of the most studied SNP of P53. Functional studies revealed that this polymorphism modifies the P53 transcriptional activity and show association with cancer susceptibility (11). Furthermore this SNP has approved effects on immune system and also chemoresistance of tumor cells (12-14).

P72 allele is significantly more common than R72 between women with recurrent implantation failure (15-17). Two most accepted explanations offered are: 1) impact of this allele on the expression levels of LIF factor and 2) effect of this allele on maternal immune system function against implanting embryo.

Several studies show that maternal immune system has an immune tolerance against implanting embryo but as has proven, P72 allele has an association with autoimmune disorders such as lupus erythematosis and arthritis rheumatoid (14). It is possible that presence of P72 allele may sensitize maternal immune system against implanting embryo and lead to embryo rejection. 

Expression analysis of cellular models bearing either of these tow alleles reveal that P72 allele induces leukemia inhibiting factor (LIF) expression two fold lower than R72 allele (18-19). As will be explained, LIF has an improved effect on the success rate of pregnancy (20).

**Leukemia inhibiting factor (LIF)**

The human LIF gene plays an essential role in embryo implantation. Expression of LIF is continuous in the uterus however it shows a transient expression peak during pregnancy and this peak coincides with the onset of implantation. Furthermore LIF^−/−^ mutation in mice leads to defect in maternal reproduction attributable to failure of implantation (9, 21-24). LIF^-/-^ mice embryo reach to the blastocyst stage but cannot implant into uterus (25-35).

p53^−/−^ mice show decreased uterine LIF levels and impaired implantation. Interestingly, LIF levels rescue via injection of exogenous LIF, leads to a significant enhancement of implantation and eventually reproduction in p53^−/−^ female mice (9, 23, 36-37). These findings suggest that mutations of P53 may lead to the absence of LIF expression following embryo implantation (36, 38, 39). In addition to p53 variants effects, LIF polymorphic alleles have also significant effect on litter size in pigs (40).

LIF overexpression in uterine secretions may be used as a potential indicator of uterine receptivity in fertile women (41-42). The majority of unexplained infertile women show significant decrease in LIF expression level, indicating the importance role of LIF in implantation (36). It has been recently identified that p53 has a specific binding site on LIF promoter and regulates both basal and inducible transcription of LIF (9, 43).

Association of LIF SNPs with fertility rate has shown that rs929271 SNP of 3UTR of LIF is significantly more common in idiopathic infertile young patients (<35years). In fertile women also this polymorphism is significantly associated with a history of infertility drugs usage. These results demonstrate an association of a SNP in the LIF gene with infertility, especially in patients under the age of 35 years (20-21, 44-45). However, several studies on the therapeutic use of recombinant LIF to enhance the success rate of IVF have failed. It seems that LIF role in human fertility is different from that in animals such as mice and further studies are needed to better understanding the importance of LIF in human reproduction and therapeutic uses of this factor.

**Other genes involved in** **p** **53 Pathway**

Recent studies suggest that several other genes involved in P53 pathway are implicated in implantation process and their polymorphisms have association with increased risk of implantation failure. 

Several studies on SNPs of P53 pathway, such as SNP309 in Mdm2, rs2279744 SNP of Mdm4 gene and rs1529916 SNP of Hausp gene has been performed (14, 20, 46, 47).

Mdm2 gene is a negative regulator of P53 and plays an important role in the regulation of mechanisms involved in implantation (14). Embryos having homozygous deletion of Mdm2 are not capable of implantation and survival. Interestingly implantation and survival ability of these embryos can be retrieved by homozygous deletion of p53 (12, 48-49). 

Interestingly, polymorphism of Mdm2 in Caucasian populations is evolutionary conserved. For example, the frequency of G allele in the Mdm2 gene in the Caucasian population is four times more than African ancestral race (14). G allele leads to higher transcriptional activity and can result in p53 diminished activity and eventually leads to a decrease in LIF gene expression. G allele of SNP309 increases the likelihood of implantation failure (10). This allele also accumulates in young patients with infertility and implantation failure (14, 50-52). 

Mdm4 is the other gene involved in P53 pathway which its polymorphism is related to increased risk of implantation failure. Mdm4 gene is structurally homologous of Mdm2 and in addition to regulating p53 gene negatively, regulates p73 gene (53, 54). Mice with homozygous deleted p73 gene were also impaired in implantation and thus infertile. T allele of Mdm4 gene rs2279744 polymorphism shows a high frequency in the population of young patients suffering from infertility and also in elderly patients with this defect. It seems that Mdm4 may regulate human fertility through p53-dependent and p53-independent pathways (14, 53).

To date only one study has been performed on the frequency of p53 gene polymorphism in Iranian patients with infertility. In this study, 70 infertile patients with recurrent implantation failure (RIF) and 32 fertile women with at least two successful pregnancies were studied. This study showed the accumulation of P72 allele in patients. However, other polymorphisms of p53 pathway in Iranian population remain to be investigated .

**P53 pathway independent genes**

In addition to p53 pathway members, there are several other genes which are implicated in embryo implantation. Majority of these genes are involved in invasion of embryo into endometrium and also in pregnancy hormonal homeostasis. 

**prostaglandin-endoperoxide synthase 2 (PTGS-2) gene**

Cox-2 enzyme encoded by PTGS-2 gene, is an inducible enzyme in prostaglandin construction pathway which is induced by a range of stimuli such as growth factors and mitogens (11).

Studies on the expression pattern of Cox isoforms in the preimplantation mouse uterus indicate the role of these enzymes in embryo implantation (11). Furthermore, studies on cox-2^-/-^ mice indicate an impaired angiogenesis into the implantation site (55). Ptgs2 mutation leads to multiple defects in the reproductive process including implantation (56). In human, expression level of COX-2 in RIF and infertile women decreases in comparison to healthy controls (57). Given these results, the association between the promoter polymorphism -765G> C and RIF were evaluated and the results showed that -765C allele is associated with increased risk of RIF (58).

**MUC-1 gene**

Transmembrane mucin-1 (MUC-1) is a glycoprotein expressed on the endometrial cell surface and can act as a barrier to implantation. During uterine receptive period, MUC-1 expression shows a dramatic decrease (55-56).

The gene that encodes this molecule is composed of a polymorphic tandem repeat of 60 nucleotides. Several studies indicate that MUC-1 genetic polymorphism is associated with implantation failure in patients with a history of recurrent abortion (55-56).

Several studies indicated that smaller alleles of MUC-1 show a higher frequency in women with infertility due to embryo implantation failure when compared to patients with no history of infertility (59-60). However, results of some other experiments suggest that there is no effect of the polymorphic MUC-1 sequence on the implantation failure (3, 61-62).

Considering the increase of MUC-1 expression in response to progesterone and also the relationship of shorter MUC-1 alleles with infertility, the anti adhesive role of MUC-1 in human becomes controversial (62, 63).

Horne et al. studies on endometrial pinopodes, using electron microscopy and immunohistochemistry confirmed that MUC-1 was linked with embryo adhesion. However they also showed that abnormal endometrial expression of MUC-1 is associated with failure of embryo implantation. This abnormal expression shows concordance with retention of nuclear progesterone receptor (PR) particularly in epithelial cells (3).

**Human progesterone receptor (hPR) gene**

Another genetic polymorphism which shows association with implantation failure risk, locates in human progesterone receptor gene. Human progesterone receptor (hPR) gene is a dual function gene which functionally encodes two different isoforms with different transcriptional factor activity, hPR-A and hPR-B (64, 65). In fact this gene is under control of two different promoters that lead to protein translation start from two distinct positions. As a result, the longer isoform, hPR-A, has 165 additional amino acid residues on its amino terminus end (66-68). The presence of this additional segment leads to the change of hPR-B conformation and significant difference between the target genes and physiologic effects of the two isoforms (66).

Isoforms deletion studies on animal models show that the imbalance between these isoforms expre--ssion leads to severe abnormalities in ovarian and uterine function and defective implantation (69).

To date several polymorphisms in hPR gene have been identified. In 1995 a small (306 bp) insertion in G intron of hPR was found and named PROGIN (70). This variant together with two other SNPs that are linked to PROGIN, V660L and H770H, were named PROGIN complex. Further-more, a fourth hPR genetic variant, +331G/A, was found that influences on hPR isoforms expression ratio (69, 71).

Pisarska et al. demonstrated that this complex has significant association with idiopathic infertility, while Cramer et al. in 2003 showed that PROGIN complex has no clear effect on implantation failure risk (69, 72). However Cramer et al.’*s *investigation results surprisingly, suggest that PROGIN complex frequency increases with the number of implantation failure (69, 73). 

**Mucin-4 (MUC-4) gene**

The most critical step in embryo implantation is adhesion of outer trophectoderm layer of the blastocyst into the luminal epithelium (74-77). This process is dependent on expression of adhesion molecules and suppression of anti adhesion molecules expression (22). Mucins are important group of adhesion molecules that show a wide range of tissue expression (78). Among mucin molecules, Muc-4 is an interesting candidate to explore because of its high expression level in endometrial epithelium (79).

Muc-4 has an important role in invasion of human cytotrophoblasts into endometrium (80). Since lubricating function of muc-4 in lubricating of reproductive tracts it hypothesized, those different-size alleles of muc-4, resulting from VNTR polymorphisms of this gene, affect receptivity of endometrium and implantation success rate (81). However Koscinski et al. findings suggest that the different-sized muc-4 alleles do not interfere with implantation (82). Interestingly, other genetic variants of muc-4 were found to be corre-lated with endometriosis related infertility (81). 

## Conclusion

According to the presented data, implanting embryo behave as a tumor against endometrium. Invasion and angiogenesis are critical steps in this process. By genotyping of RIF suffered couples we can predict the risk of IVF failure and present appropriate therapeutic options.
